# AQUEDUCT Intervention for Crisis Team Quality and Effectiveness in Dementia: Protocol for a Feasibility Study

**DOI:** 10.2196/18971

**Published:** 2020-10-13

**Authors:** Emma Elizabeth Broome, Donna Maria Coleston-Shields, Tom Dening, Esme Moniz-Cook, Fiona Poland, Miriam Stanyon, Martin Orrell

**Affiliations:** 1 National Institute for Health Research Nottingham Biomedical Research Centre Hearing Sciences, Division of Clinical Neuroscience University of Nottingham Nottingham United Kingdom; 2 Division of Psychiatry & Applied Psychology, Institute of Mental Health, School of Medicine University of Nottingham Nottingham United Kingdom; 3 Faculty of Health Sciences University of Hull Hull United Kingdom; 4 School of Health Sciences University of East Anglia Norwich United Kingdom

**Keywords:** dementia, crisis, mental health, community services, feasibility study

## Abstract

**Background:**

Specialist community teams often support people with dementia who experience crisis. These teams may vary in composition and models of practice, which presents challenges when evaluating their effectiveness. A best practice model for dementia crisis services could be used by teams to improve the quality and effectiveness of the care they deliver.

**Objective:**

The aim of this study is to examine the feasibility of conducting a large-scale randomized controlled trial comparing the AQUEDUCT (Achieving Quality and Effectiveness in Dementia Using Crisis Teams) Resource Kit intervention to treatment as usual.

**Methods:**

This is a multisite feasibility study in preparation for a future randomized controlled trial. Up to 54 people with dementia (and their carers) and 40 practitioners will be recruited from 4 geographically widespread teams managing crisis in dementia. Quantitative outcomes will be recorded at baseline and at discharge. This study will also involve a nested health economic substudy and qualitative research to examine participant experiences of the intervention and acceptability of research procedures.

**Results:**

Ethical approval for this study was granted in July 2019. Participant recruitment began in September 2019, and as of September 2020, all data collection has been completed. Results of this study will establish the acceptability of the intervention, recruitment rates, and will assess the feasibility and appropriateness of the outcome measures in preparation for a large-scale randomized controlled trial.

**Conclusions:**

There is a need to evaluate the effectiveness of crisis intervention teams for older people with dementia. This is the first study to test the feasibility of an evidence-based best practice model for teams managing crisis in dementia. The results of this study will assist in the planning and delivery of a large-scale randomized controlled trial.

**International Registered Report Identifier (IRRID):**

DERR1-10.2196/18971

## Introduction

### Background

Dementia is a progressive condition that affects over 850,000 people in the United Kingdom [1]. The cognitive, behavioral, and psychological symptoms of dementia include memory loss, changes in reasoning and planning skills, and communication difficulties that may impair ability to perform daily activities [2]. Improving dementia care remains a key priority from the Prime Minister’s Challenge on Dementia [3]. Community support, with the aim of reducing hospital admissions, is one way to respond to this policy initiative; however, fluctuations in the health and social circumstances of the person with dementia may cause a breakdown in care which could lead to crisis.

A dementia mental health crisis can be defined as “a need for urgent mental health assessment and intervention for people with dementia who live in the community” (from Hoe J, Ledgerd R, Devine M, Toot S, Challis D, Orrell M. Home Treatment Manual 2012 Version 4. Support at Home: Interventions to Enhance Life in Dementia (SHIELD); unpublished). Crisis situations in dementia are common and often result in a hospital admission. Risk factors for breakdown of care at home include increased carer burden and inadequate social support [4]. Increased contact with general practitioners and case manager consultations are recommended to help manage instances of crisis [5]. Support for people with dementia and their carers at a time of crisis is often managed through secondary mental health services. These services involve teams that vary in name and composition and may include crisis resolution and home treatment teams and dementia rapid response teams, though in some instances, there are no suitable services. Some teams may be commissioned to specifically provide support for older people, while others may be nonage-defined. From here on in, these services will be referred to as *teams managing crisis in dementia*. A recent scoping survey [6] highlighted the disparity in services across England; teams managing crisis in dementia vary in terms of name, set-up, delivery, policy, and procedures. Further high-quality evidence is required to support the effectiveness of teams managing crisis in dementia in reducing hospital admissions and preventing breakdown of care at home, and to improve knowledge on how teams can be supported to deliver care for people with dementia in crisis.

### Preliminary Work

The Achieving Quality and Effectiveness in Dementia Using Crisis Teams (AQUEDUCT) program comprises 3 work packages following the Medical Research Council’s Framework for the Development and Evaluation of Randomized Controlled Trials for Complex Interventions [7]. The first work package (WP1) consisted of 2 strands: a systematic literature review to examine the effectiveness of crisis interventions for older people and a scoping review to map and understand operational procedures in current services [6], and subsequently, qualitative work (including interviews; focus groups; consultations; and a consensus conference involving people with dementia, carers, practitioners, and stakeholders) was used to identify and establish agreement about key elements of best practice in teams managing crisis in dementia.

The strands from WP1 contributed to the development of a model of best practice comprising 50 best practice statements, a best practice tool, and a resource kit [8], after which, 12 teams managing crisis in dementia and 5 noncrisis older adult mental health teams field-tested the AQUEDUCT best practice tool and resource kit. The feedback from these teams was used to amend the resource kit for future use in the second work package (WP2)—the feasibility study. Findings from this feasibility study will be used to inform the future large-scale randomized controlled trial in the third work package (WP3). This paper describes the protocol for WP2 only.

### Aims and Objectives

The aims of WP2 of the AQUEDUCT research program were to (1) conduct a feasibility study of use of the resource kit in relation to practice, care outcomes, and costs; (2) gather feedback from participants about the acceptability and feasibility of the research procedures; and (3) refine the resource kit for use in the randomized controlled trial in order to (1) determine the feasibility of recruitment to a large-scale randomized controlled trial; (2) refine the eligibility criteria for teams managing crisis in dementia for a future definitive randomized controlled trial; (3) determine the relevance and acceptability to National Health Service (NHS) practitioners; (4) determine the acceptability to people with dementia, carers, and NHS practitioners of the trial procedures; (5) assess the ability of the NHS sites to implement the resource kit; (6) assess the training and support needs for NHS practitioners using the resource kit; (7) evaluate resource kit uptake and fidelity when used through NHS services; (8) assess follow-up and outcome completion rates; (9) determine the relevance and acceptability of a range of outcome measures to inform selection of the primary outcome for the main trial; and (10) evaluate the utility and acceptability of resource use questionnaires for use in an economic evaluation in a future randomized controlled trial.

**Figure 1:**
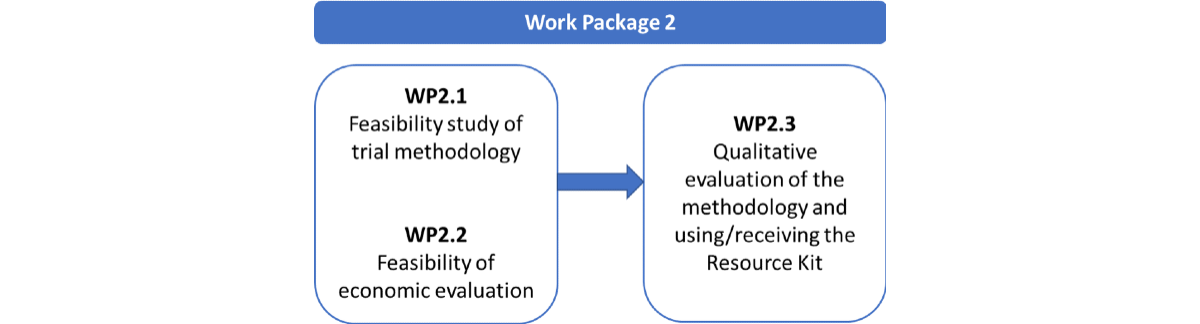
Design and components of WP2.

The design of WP2 is illustrated in Figure 1. The main component—WP2.1—is the overall feasibility study; WP2.2 will examine the feasibility of an economic evaluation of the resource kit, and WP2.3 is a qualitative evaluation of the experience of using the resource kit and its acceptability.

## Methods

### Site Selection

This is a multisite pre–post feasibility study with all sites allocated to the AQUEDUCT resource kit intervention. Recruitment will take place at 4 sites which will be purposively selected from across England to ensure a diverse range of teams managing crisis in dementia models and service user demographics. The number of teams approached and reasons for teams declining to participate will be documented. This will allow structural or process issues which influence participation to be identified and considered for how they may impact site selection in the main trial.

### Criteria

Inclusion criteria will be as follows: (1) teams managing mental health crises in dementia in community settings, (2) people with dementia receiving input from the teams managing crisis in dementia during the team’s use of the resource kit, and (3) carers providing support for a person with dementia in receipt of input (using the resource kit) from the teams managing crisis in dementia.

Exclusion criteria will be as follows: (1) team is not defined by NHS Trust as having a role in dementia mental health crisis management, (2) team does not meet the following definition for mental health crisis management—providing urgent mental health assessment and intervention for people with dementia in the community, and (3) team is not able to demonstrate capacity and capability to complete required research activities.

### Participant Recruitment

This study will recruit 3 different groups of participants: teams managing crisis in dementia practitioners, people living with dementia, and carers. Recruitment will take place in different ways. All participants will be asked to consent separately, by signing relevant consent forms for each stage of the research with which they wish to engage. This section presents the recruitment pathway for WP2.1, the main feasibility study. Recruitment for WP2.2 and WP2.3 will be outlined later in the protocol.

Practitioners will be identified and recruited from participating teams managing crisis in dementia. Once the NHS Trust has formally agreed to participate in the study, each team manager or senior practitioner will receive an information pack which will include an information sheet for potential practitioner participants. The team manager will identify 2 practitioners who will act as research coordinators for the site; these practitioners will be given up to 3 days to decide whether or not they wish to participate. A member of the AQUEDUCT research team will then conduct a site set-up visit to answer any questions and seek written informed consent. From this point, the research coordinators will be responsible for arranging and confirming consent with other practitioners at the site. All teams will complete good clinical practice [9] training before their study start date.

People with dementia and carers who are referred to the teams managing crisis in dementia caseload during the first 2 weeks of each team's implementation of the resource kit will be identified and recruited by participating practitioners. Potentially eligible participants will be approached by the practitioner who will give them an appropriate information sheet and explain to them that the team managing crisis in dementia has agreed to participate in the AQUEDUCT research program. Potential participants will be given up to 3 days to decide whether or not they wish to participate; if in agreement, they will then sign a consent form.

Where a carer also agrees to participate in the study, the carer will be asked to provide written informed consent for their own participation in the feasibility study. A carer’s decision to participate or not will not affect the involvement of a consenting person with dementia.

### Sample Size

The aim of this feasibility study is to estimate rates of recruitment and completion rates, and to refine eligibility criteria and other research procedures; therefore, no formal sample size calculation is required. The proposed sample size includes a total of 40 teams managing crisis in dementia practitioners and 54 people with dementia and carers across the 4 NHS sites; this is considered sufficient to establish feasibility and to inform the future large-scale randomized controlled trial.

### Ethics and Mental Capacity

Ethical approval was given by the West Midlands–Coventry and Warwickshire Research Ethics Committee (19/WM/0132) on July 14, 2019 and Health Research Authority approval was given on July 15, 2019. The study sponsor is Nottinghamshire Health care NHS Foundation Trust.

On first contact with the person with dementia, and at every subsequent meeting with a person with dementia, the teams managing crisis in dementia practitioner will determine the mental capacity (according to the Mental Capacity Act 2005 [10]) of the person with dementia to give informed consent to take part in the research. Where the person with dementia is thought not to have the mental capacity to give informed consent, the view of the person with dementia’s carer will be taken into account as they will have a greater knowledge of the person with dementia’s abilities over time. The carer consultee will be asked to consider what would be in the best interests of the person with dementia according to their previous or currently expressed views. In these instances, the carer will be provided with a consultee information sheet and a carer consultee declaration form.

### Withdrawal

People with dementia can withdraw from the study at any time without any impact on their current or future care. Carers may also withdraw from the study, and this will not affect the person with dementia’s continued involvement in the study. Participant information sheets and consent forms will inform participants of their right to withdraw from the research for any reason (which does not need to be stated) and at any time, without any effect on their employment (for teams managing crisis in dementia practitioners) or input from services (for people with dementia and carers).

### Patient and Public Involvement

Throughout the development of the AQUEDUCT research program, the research team has extensively consulted with people with dementia and their carers. Patient and public involvement was integrated throughout WP1, to inform development of the AQUEDUCT resource kit. The protocol for WP2 has been developed in consultation with the AQUEDUCT patient and public involvement reference group, and all study documentation and participant recruitment procedures have been reviewed by patient and public involvement representatives.

### AQUEDUCT Intervention

The AQUEDUCT resource kit is an online resource for teams managing crisis in dementia, designed to assist teams in evaluating and improving their practice according to the best practice model developed in WP1 of the research program. The resource kit comprises 3 components: the best practice tool which enables teams managing crisis in dementia to evaluate their practice according to 50 best practice statements, the home treatment package developed during the National Institute for Health Research (NIHR)–funded Support at Home—Interventions to Enhance Life in Dementia study (from Hoe J, Ledgerd R, Devine M, Toot S, Challis D, Orrell M. Home Treatment Manual 2012 Version 4. Support at Home: Interventions to Enhance Life in Dementia (SHIELD); unpublished), and a collection of templates and documents that can be used directly or adapted by teams to suit their practice.

At the set-up visit, or subsequently, participating practitioners will complete online training on use of the AQUEDUCT resource kit. They will then complete a posttraining self-assessment to provide information about the effectiveness of the online training. The self-assessment consists of multiple-choice questions that assess the ability, training, and support needs of NHS practitioners using the resource kit. The aim of this assessment is to identify any misunderstandings or areas that require further explanation. Topics focus on study procedures and the best practice toolkit.

For the purpose of the feasibility study, each team will be given 3 weeks to complete the best practice tool before the implementation phase, to determine areas in which the teams managing crisis in dementia could improve practice. The team will then implement relevant elements of the resource kit that will assist them in improving practice during an 8-week implementation phase. The team will recomplete the best practice tool at the end of the implementation phase.

The AQUEDUCT research team will have weekly contact with team practitioners to obtain feedback and provide support for implementation of the resource kit. Support elements will be monitored by the research team to identify usage and costs. teams managing crisis in dementia practitioners will maintain activity records to monitor time spent implementing the resource kit in practice during the implementation phase.

### Study Outcomes

#### Feasibility Study Outcomes (WP2.1)

One of the main aims of the feasibility study is to determine the most suitable outcome measures for the proposed randomized controlled trial. The primary outcome measure to be used in the main trial will be selected from among those described below, based on relevance and acceptability in this feasibility study.

#### Outcome Measures for the Person With Dementia

Both the self-completed and proxy versions of the Dementia Quality of Life Questionnaire [11], measuring quality of life for people with dementia, will be used. The Client Satisfaction Questionnaire [12], measuring user satisfaction of service received; the Neuropsychiatric Inventory [13], a carer-completed measure that assesses neuropsychiatric symptoms for the person with dementia rating the frequency of the symptoms on a 4-point Likert scale, and severity on a 3-point scale; and the Bristol Activities of Daily Living Scale, a carer-rated measure that assesses daily living activities [14] will also be used.

#### Outcome Measures for the Carer

European Quality of Life 5 Dimensions questionnaire [15], measuring health-related quality of life; Hospital Anxiety and Depression Scale, a self-completed measure that assesses anxiety and depression [16]; the Neuropsychiatric Inventory severity of symptoms (3-point scale) and the impact of symptoms manifestations (5-point scale) to determine caregiver distress associated with neuropsychiatric symptoms [13]; and Client Satisfaction Questionnaire [12] will be used.

#### Outcome Measures for Teams Managing Crisis in Dementia

The following data will be collected: initial best practice tool score for the teams managing crisis in dementia; final best practice tool score for the teams managing crisis in dementia; number of hospital admissions for the teams managing crisis in dementia over the study period; total number of referrals received by the teams managing crisis in dementia over the study period (to include nondementia referrals); number of specific dementia crisis referrals received by the teams managing crisis in dementia over the study period; number of inappropriate referrals to the teams managing crisis in dementia over the study period; teams managing crisis in dementia practitioner absenteeism over the study period; total number of hospital beds available to the service or organization during the study period; and number of hospital beds available to the teams managing crisis in dementia over the study period.

#### Health Economic Outcomes (WP2.2)

This substudy will test the feasibility of conducting a full economic evaluation of the resource kit in the future randomized controlled trial. The aim will be to test the relevance and acceptability to people with dementia and carers of the questionnaires to be considered for the full economic evaluation in the large-scale randomized controlled trial. It will include at least 4 carers of people with dementia recruited by each of the 4 teams to reflect living arrangements such as a spouse living with a person with dementia or an adult-child living elsewhere. It will involve an analysis of the specific cost of the resource kit, identify appropriate sources of data to be used in the full economic evaluation, and how best to collect these data.

To assess the feasibility of the economic evaluation, carers who are taking part in WP2.1 will be approached by team practitioners and asked if they will consider participating in the feasibility study of the economic evaluation as well. If agreeable, potential participants will then be contacted by a member of the AQUEDUCT research team and will be provided with an information sheet, given an opportunity to ask questions, and given up to 3 days to decide whether or not they wish to participate in this part of the research. If they wish to be involved, participants will sign the relevant consent form.

To calculate the cost of the resource kit, the following components will be considered: (1) the cost of producing and maintaining the resource kit, including the cost of producing and maintaining the best practice tool, all resource materials in the resource kit, all guidance and explanatory information, and the website which supports the resource kit and (2) the cost of skills training for teams managing crisis in dementia practitioners in the use of the resource kit, including all training materials.

It will be assumed that, in the large-scale randomized controlled trial, the lifetime incremental cost per quality-adjusted life year gained for the resource kit versus treatment as usual will be estimated from NHS and Personal Social Services perspectives and from a societal perspective. The resources used and unit cost data for these components will be collected through a modified version of the Clinical Service Receipt Inventory [17]. Potential sources of health-related quality of life data suitable for estimating quality-adjusted life years for people with dementia and carers will also be collected.

#### Qualitative Evaluation of Participant Experience (WP2.3)

This part of the study will evaluate the experience of applying the research methodology and the acceptability and relevance to participants of using or receiving input from the teams managing crisis in dementia with the resource kit. Findings from the qualitative substudy will be used to modify the resource kit and research methods to be used in the large scale randomized controlled trial. Team practitioners who were involved in the main part of the feasibility study will be approached by a member of the AQUEDUCT research team to invite them to participate in the qualitative evaluation. Potential participants will be given a relevant information sheet, an opportunity to ask questions, and up to 3 days to decide whether or not they wish to participate in advance of giving consent.

All potentially eligible people with dementia and carers will be provided with a relevant information sheet; the research will be explained to them verbally, and potential participants will have the opportunity to ask questions and will be given up to 3 days to decide whether or not they wish to participate in advance of giving consent.

Once informed consent has been provided, team practitioners, people with dementia, and carers will complete bespoke questionnaires to explore their perspectives of how the research procedures were applied and of the acceptability and relevance of using, or receiving input on, the resource kit. Practitioners will be asked to answer statements such as “Was the Best Practice Toolkit training an appropriate length of time?’” with either yes, somewhat, or no. The questionnaire also includes some open-ended questions such as “If you answered no, please provide further details.” The framework for the bespoke questionnaires is outlined Textbox 1.

**Framework for qualitative substudy.**

Experience of

- online training and related activities (teams managing crisis in dementia only)
- completing the best practice tool (teams managing crisis in dementia only)
- recruiting people with dementia or carers and of being recruited (all participants)
- using the resource kit (teams managing crisis in dementia only)
- completing other research-related activity (teams managing crisis in dementia only)
- completing the questionnaires (all relevant participants)
- contact with the AQUEDUCT research team (all relevant participants)
- clinical team input (people with dementia and carers only)

### Data Collection

Table 1 outlines procedures and assessments at each time point during the study. Upon recruitment, team practitioners will complete a demographic information sheet. Each team will complete the best practice tool at 2 time points: baseline (preimplementation) and study close (postimplementation). The qualitative questionnaires will be completed by team practitioners at the end of the 8-week implementation phase.

**Table 1:** Study procedures for WP2.

Assessments and procedures			Study set-up (3 weeks)	Baseline	Implementation phase (8 weeks)	Study close (2 weeks)
**WP2.1^a^**						
	**Practitioners**					
		Informed consent	X			
		Demographic information	X			
		Best practice tool intervention	X			X
	**People with dementia**					
		Eligibility screen		X		
		Informed consent		X		
		Demographic information		X		
		Dementia Quality of Life Questionnaire		X	X	
		Client Satisfaction Questionnaire			X	
	**Carers**					
		Eligibility screen		X		
		Informed consent		X		
		Demographic information		X		
		European Quality of Life 5 Dimensions Questionnaire		X	X	
		Hospital Anxiety and Depression Scale		X	X	
		Client Satisfaction Questionnaire		X	X	
		Bristol Activities of Daily Living Scale		X	X	
		Dementia Quality of Life Questionnaire-Proxy		X	X	
		Neuropsychiatric Inventory symptom frequency and severity		X	X	
**WP2.2**						
	**Carers**					
		Informed consent		X		
		Demographic information		X		
		Client Service Receipt Inventory		X		X
		Experience questionnaire				X
**WP2.3**						
	**Practitioners**					
		Informed consent				X
		Demographic information				X
		Experience questionnaire				X
	**People with dementia and carers**					
		Informed consent				X
		Demographic information				X
		Experience questionnaire				X

^a^WP: work package.

Data will be collected at 2 time points for people with dementia and carers. Upon recruitment, demographic information and baseline questionnaires will be completed; follow-up questionnaires will be completed at the end of the period during which the person with dementia and the carer has received team input. Participants not included in the study will be recorded on the Person with Dementia and Carer data summary sheet. The reason for noninclusion or the reason for declining to participate will be recorded where possible.

### Data Management

Upon signing a consent form, all participants will be allocated a unique identification code to ensure anonymity. Individual participant information obtained as a result of this research will be considered strictly confidential. Confidentially will be maintained through ongoing use of unique identifiers. All data will be treated as confidential and the NHS Code of Confidentiality [18], 2016 General Data Protection Regulation [19], and 2005 Good Clinical Practice guidelines [9] will be adhered to. Insurance and indemnity arrangements will be covered by the study sponsor (Nottinghamshire Health care NHS Foundation Trust).

### Data Analysis

Data analysis to address the feasibility aims of this study will be primarily descriptive. Feasibility outcomes will be estimated using descriptive statistics (with 95% confidence intervals) and will consider recruitment rates, retention rates, amount of missing data, and intervention adherence. The rate of protocol adherence will be reported in terms of participants (practitioners, people with dementia, and carers) who adhere to the required research activities. Key characteristics (personal, demographic and, where appropriate, clinical information from the case report form) will be compared between participants and those who are ineligible or who do not consent to take part, to ascertain adequacy of inclusion and exclusion criteria and likely generalizability of this research to the required targeted populations. The same characteristics will be compared between those who complete all required research activities and those who do not.

Process data will also be collected by the teams managing crisis in dementia and the AQUEDUCT research team. This will include the number of sites approached, the number of participants approached as well as the number recruited, and reasons for not participating given by those who decline to participate. The teams managing crisis in dementia will also record time spent on research activities and on implementing the resource kit.

### Adverse Events

No adverse events are anticipated. All adverse events will be recorded in the case report form and the Person with Dementia and Carer data summary sheet. Participants will be informed that they can stop their participation at any time without any impact on their employment or clinical input.

## Results

Ethical approval for this study was granted in July 2019. Participant recruitment began in September 2019, and as of September 2020, all data collection has been completed.

## Discussion

This protocol describes a multisite feasibility study of an evidence-based best practice model for teams managing crisis in dementia. Previous research revealed only limited evidence in support of crisis teams reducing hospital admission rates, and despite an increase in the number of published studies, their designs remain methodologically limited [6]. The AQUEDUCT resource kit addresses the current care gap by providing more robust evidenced-based dementia crisis care model. This study will add to knowledge concerning the feasibility and acceptability of implementing a model of best practice for teams managing crisis in dementia. Data gathered in this study will also provide an opportunity to modify the AQUEDUCT resource kit.

We anticipate that this information can be used to improve the quality and effectiveness of crisis teams that work with people with dementia. If the AQUEDUCT intervention is found to be effective, this program could influence how services are organized, with benefits for people with dementia and their carers. The long-term impact of a standardized model of dementia crisis care working may be to reduce hospital admissions and to reduce care costs. These impacts may improve quality of life for people with dementia and carers.

## Appendix

Multimedia Appendix 1 Peer-review reports.

## Abbreviations

AQUEDUCT: Achieving Quality and Effectiveness in Dementia Using Crisis Teams
NHS: National Health Service
NIHR: National Institute for Health Research
WP: work package
