# Supplementary material for: AQUEDUCT Intervention for Crisis Team Quality and Effectiveness in Dementia: Protocol for a Feasibility Study
Source: JMIR Res Protoc. 2020 Oct 13;9(10):e18971. doi: 10.2196/18971 (PMC7592065; doi:10.2196/18971)
Supplement: Multimedia Appendix 1 [file resprot_v9i10e18971_app1.pdf]

PGfAR Competition 13 Stage 2

Peer Review Form

**Application Reference**	RP-PG-0612-20004
**Application Title**	Achieving Quality and Effectiveness in Dementia Using Crisis Teams (AQUEDUCT)
**Applicant**	Professor Martin Orrell
**Applicant Organisation**	University College London
**Total Amount Requested**	£1,978,647.00
**Reviewer Number**	1

**Reviewer Expertise**	Other (please see below)<br>Health Psychology - Quality of Life in Dementia

1. RELEVANCE of the proposed research

a) How relevant and important is the proposed research to the priorities and needs of the NHS, and does it offer a health/healthcare solution with demonstrable benefit to patients?
b) Does the application demonstrate an awareness and understanding of previous relevant research or developments in this area?
c) To what extent does the proposed research add distinct value or provide an advance to what is already known from other work currently in progress or already completed in this area?

The proposed research is particularly relevant given the need to explore non-drug interventions for people with dementia alongside home-based care. The research is built on solid foundations and adds to an existing programme of research.

2. QUALITY of the proposed work

2a. Research design

a) Is the proposed research of high quality and does it address the stated objectives?
b) How convincing and coherent is the proposed rationale and approach?
c) Is the proposed design and methodology for all elements of the research well defined, appropriate, valid, robust and feasible within the timeframe and resources requested?
d) What are the strengths and weaknesses of the research design as proposed?

The proposed study is of high quality and adequately meets the main objectives. The methodology is well defined and has no obvious weaknesses.

2. QUALITY of the proposed work

2b. Work plan and proposed management arrangements

a) Do the work plan and project management arrangements give confidence that proposed milestones will be met within the specified timeframe?

b) Can you identify any difficulties that the applicants are likely to encounter in meeting their milestones?
c) Have the major ethical, scientific, technical and organisational challenges, as well as any issues around intellectual property rights, been identified and will they be addressed adequately?
d) Are the necessary clinical, academic or organisational links needed to support the research, or help translate it into practice, in place?
e) Is long-term, large scale programme grant funding appropriate; is there added value over and above the dividends from the individual elements?

The workplan seems entirely reasonable and project milestones are achievable.

---

3. STRENGTH of the research team

a) Are the applicants familiar with the methodologies outlined in their application and are they well qualified to undertake the proposed work on the basis of track record in relevant areas (as judged by publication output and previous research funding)?
b) Does the research team have the necessary breadth and depth of expertise to deliver the planned work?
c) Are the roles of the team members clearly described, and is the overall team well coordinated?
d) If the lead applicant is inexperienced, does he/she have appropriate support (e.g. from their host institution and more senior colleagues) to deliver the work plan?

This appears to be a very strong research team and all aspects of the study are well-supported.

---

4. IMPACT of the proposed work

a) Have the applicants outlined an appropriate and adequate approach to disseminating the result of their research (including engaging with healthcare planners and/or policy makers)? Could this be developed further?
b) Considering the plans for dissemination, what is the likelihood of a significant benefit to the NHS and patients within 3-5 years of the end of the funding period?
c) Have the applicants identified any new intellectual property that will be produced during the course of this research and are there plans to protect and exploit it?

This project could have significant impact for people with dementia, carers and costs of care. Through the use of the AQUEDUCT website, resources can be made available quickly and efficiently and I would envisage that the NHS would see a significant benefit soon after project completion.

---

5. VALUE for money (justification for proposed costs)

a) Are the resources required for this research, including staffing, clearly justified? Are they essential for the work proposed?
b) Where the application offers arguments about financial benefits, please assess how realistic these might be.
c) Taking into account the expected benefits of the work proposed and the level of resources requested, does this application represent good value for money?
d) If required, are funds requested for Support and Treatment Costs appropriate?

The costs appear reasonable - no further comment

---

6. INVOLVEMENT of patients and the public

Where applicable (N.B. if there is no patient and public involvement, please address question d below)
a) What is your assessment of the patient and public involvement (if any) in the development of the application including involvement in: identifying the research topic; prioritising the research questions; preparing the application (e.g. contributing to the research design)?
b) What is your assessment of any proposed plans for patient and public involvement throughout the duration of the research? Can you identify particular strengths, weaknesses and/or areas for improvement?
c) Are the resources set aside for patient and public involvement appropriate - including plans for a

training and support budget?
d) If there is no patient and public involvement in the application, what is your assessment of the reasons given for this?

PPI has been adequately addressed and appears throughout this study

7. Additional comments

Do you have any other comments or suggestions for how the proposed research might be improved? If so, please indicate whether you see these as critical factors.

I thought this was an excellent proposal and can see no significant weaknesses.

PGfAR Competition 13 Stage 2

Peer Review Form

**Application Reference**	RP-PG-0612-20004
**Application Title**	Achieving Quality and Effectiveness in Dementia Using Crisis Teams (AQUEDUCT)
**Applicant**	Professor Martin Orrell
**Applicant Organisation**	University College London
**Total Amount Requested**	£1,978,647.00
**Reviewer Number**	2

**Reviewer Expertise**	Researcher in the same/a very similar field<br>Doctor of Education. Fellow of the Higher Eduction Academy. Course leader, MSc Dementia Studies. NIHR grant holder.

1. RELEVANCE of the proposed research

a) How relevant and important is the proposed research to the priorities and needs of the NHS, and does it offer a health/healthcare solution with demonstrable benefit to patients?
b) Does the application demonstrate an awareness and understanding of previous relevant research or developments in this area?
c) To what extent does the proposed research add distinct value or provide an advance to what is already known from other work currently in progress or already completed in this area?

Keeping people with dementia in their own homes for as long as possible is a high priority for the NHS. Unnecessary moves to hospital or formal care are not in the best interests of service users with dementia, nor are they cost-effective or a good use of resources. Having said this, it would have been helpful to see some further definition of what constitutes a crisis for purposes of this proposal. In some cases it is right and necessary to admit a person with dementia to hospital (eg acute physical illness or accident), therefore there is not necessarily a correlation between quality of service and rate of hospital admission. Clearly there are many situations where it is worse for a person with dementia to be neglected by services and left without treatment in his/her own home. It may be that the desired reduction in hospital admissions is intended to relate exclusively to psychiatric admissions, but this is not argued consistently throughout. I would question the emphasis on reduction in hospital admissions as the primary outcome measure, ie will any form of practice leading to a reduction in hospital admissions therefore be classed as 'best practice'?

There is also a slight logical inconsistency regarding value-added, in that the applicants estimate that where Dementia Crisis Teams are in place this reduces hospital admissions by 30%, ie to 70% of the otherwise expected rate of admission. They then suggest that the implementation of the Resource Kit might be expected to reduce this by a further 20%, ie to 50% of the expected rate. Logically this would suggest that - despite their deficiencies - more value is added by establishing DCTs in areas that do not yet have them, than in implementing the RK in those areas where DCTs already exist.

2. QUALITY of the proposed work

2a. Research design

a) Is the proposed research of high quality and does it address the stated objectives?
b) How convincing and coherent is the proposed rationale and approach?
c) Is the proposed design and methodology for all elements of the research well defined, appropriate, valid, robust and feasible within the timeframe and resources requested?
d) What are the strengths and weaknesses of the research design as proposed?

The proposal links together a WP (1) to find out what is currently happening about crisis management in dementia care, and a proposal to change what is happening by means of an intervention which has already been identified (WP 2 and 3). Whilst the two components may be valid independently they do not fit together well methodologically.

As suggested in stage 1 feedback, existing DCTs are presented here as both the problem (patchy practice, piecemeal solutions, no standard model based on evidence etc) and as the solution, ie the source from which 'best practice' advice will emerge. It isn't very clear what criteria will be used to discern such good practice as currently exists in DCTs.

It is easy to see how the scoping exercise in WP1 will elicit a large volume of rich ethnographic data related to problems and obstacles in implementing good practice in crisis management in dementia, but much less easy to see how this will contribute to the content of a 'best practice' Resource Kit which addresses these problems. While the ethnographic data from WP1 would be excellent for a different kind of qualitative study, ie one designed simply to find out about the experiences of DCT practitioners, it doesn't fit well methodologically with the development of a resource intended to bring about quantitative changes in practice, as in WP2.

Moreover, in WP3 the nature of the intervention has already been decided and is therefore not open to the kind of emergent approach that might usually result from a qualitative, exploratory study by identifying what practitioners themselves feel is lacking, and what format they think support should be available in.

The proposed RCT is necessarily a single, rather than double, blind study in that the researcher collecting the data will be blind to which arm of the study is which, but half the participants, once recruited, will quickly realise that they are part of the control group. They will thus become aware at an early stage that they are receiving no reward for their involvement in terms of resources to enhance their service provision. This may look like a poor deal for the control group participants, and their enthusiasm for taking part may dwindle as a result.

The RK is to be compared with 'treatment as usual' only; there is no proposal to compare the RK with any other form of service support or intervention. Therefore, even if the findings are positive in favour of the RK, this will only be by comparison with a current level of service which the researchers themselves say is deficient in many ways.

---

2. QUALITY of the proposed work

2b. Work plan and proposed management arrangements

a) Do the work plan and project management arrangements give confidence that proposed milestones will be met within the specified timeframe?
b) Can you identify any difficulties that the applicants are likely to encounter in meeting their milestones?
c) Have the major ethical, scientific, technical and organisational challenges, as well as any issues around intellectual property rights, been identified and will they be addressed adequately?
d) Are the necessary clinical, academic or organisational links needed to support the research, or help translate it into practice, in place?
e) Is long-term, large scale programme grant funding appropriate; is there added value over and above the dividends from the individual elements?

The project timescale and milestones seem well-organised, and appropriate support appears to be in place. As above, I would be concerned about delay and data loss from participants who realise they are part of the control arm.

Given the extremely sensitive issues that might arise in crisis situations for people with dementia and carers there could be more evidence of having thought through ethical issues, beyond initial capacity and consent. Other relevant issues include the intrusiveness of the study taking place in

participants' own homes under emotionally difficult circumstances, and the ethical issues that might arise if the researchers feel that a crisis is being mismanaged by the DCT in question. There needs to be some statement about how this would be reported and to whom.

---

3. STRENGTH of the research team

a) Are the applicants familiar with the methodologies outlined in their application and are they well qualified to undertake the proposed work on the basis of track record in relevant areas (as judged by publication output and previous research funding)?
b) Does the research team have the necessary breadth and depth of expertise to deliver the planned work?
c) Are the roles of the team members clearly described, and is the overall team well coordinated?
d) If the lead applicant is inexperienced, does he/she have appropriate support (e.g. from their host institution and more senior colleagues) to deliver the work plan?

Yes - this is a strong and experienced team with a good record of previous funding and outputs.

---

4. IMPACT of the proposed work

a) Have the applicants outlined an appropriate and adequate approach to disseminating the result of their research (including engaging with healthcare planners and/or policy makers)? Could this be developed further?
b) Considering the plans for dissemination, what is the likelihood of a significant benefit to the NHS and patients within 3-5 years of the end of the funding period?
c) Have the applicants identified any new intellectual property that will be produced during the course of this research and are there plans to protect and exploit it?

I note that following stage 1 feedback the HTP, originally the subject of the proposed trial, has been incorporated into a more extensive proposed Resource Kit for DCTs. The outline components of the RK are specified in the revised proposal, and arrangements for conducting a feasibility study and pilot prior to the full RCT are discussed as WP2. There seems, however, to be a logical discontinuity between the scoping exercise in WP1 and the work to be undertaken in WP2. Surely, the best people to advise on the content of the RK, if it is to be useful, are the service users and carers who are the experts by experience on current crisis management, rather than the DCT teams who are delivering the current 'muddle'. My suspicion is that the content of the RK will turn out to be the same regardless of WP1, ie common sense advice that could have been derived directly from PPI advisers. If so, this would suggest that the same impact could be achieved more quickly and at less expense.

Also, in stage 1 feedback it is noted that the dissemination plans are written as though the outcome of the RCT is already known and that the findings are positive. Although I did not see the previous version, this still appears to be the case, at least in the lay summary.

---

5. VALUE for money (justification for proposed costs)

a) Are the resources required for this research, including staffing, clearly justified? Are they essential for the work proposed?
b) Where the application offers arguments about financial benefits, please assess how realistic these might be.
c) Taking into account the expected benefits of the work proposed and the level of resources requested, does this application represent good value for money?
d) If required, are funds requested for Support and Treatment Costs appropriate?

There are fewer WPs specified for this proposal than in previous ones I have reviewed, and (as above) it is questionable whether they are all necessary, or logically follow on from each other.

---

6. INVOLVEMENT of patients and the public

Where applicable (N.B. if there is no patient and public involvement, please address question d below)
a) What is your assessment of the patient and public involvement (if any) in the development of the

application including involvement in: identifying the research topic; prioritising the research questions; preparing the application (e.g. contributing to the research design)?
b) What is your assessment of any proposed plans for patient and public involvement throughout the duration of the research? Can you identify particular strengths, weaknesses and/or areas for improvement?
c) Are the resources set aside for patient and public involvement appropriate - including plans for a training and support budget?
d) If there is no patient and public involvement in the application, what is your assessment of the reasons given for this?

The direct involvement of people with dementia themselves could be more clearly specified. It is stated that they have been involved in the preparation of the proposal and that they will have an ongoing role, but there is not much detail of how this has been, or will be, facilitated or what emerged from consulting them, as distinct from the carers involved. Involvement of carers/former carers is good, including a co-applicant.

---

7. Additional comments

Do you have any other comments or suggestions for how the proposed research might be improved? If so, please indicate whether you see these as critical factors.

In one or two places I noticed that the proposal refers to managing people with dementia, rather than managing crisis situations; this isn't service user-friendly terminology and should be changed, but is not critical.

PGfAR Competition 13 Stage 2

Peer Review Form

**Application Reference**	RP-PG-0612-20004
**Application Title**	Achieving Quality and Effectiveness in Dementia Using Crisis Teams (AQUEDUCT)
**Applicant**	Professor Martin Orrell
**Applicant Organisation**	University College London
**Total Amount Requested**	£1,978,647.00
**Reviewer Number**	3

**Reviewer Expertise**	Researcher in a broadly related field<br>Health Economics, health policy; some experience in research in dementia

1. RELEVANCE of the proposed research

a) How relevant and important is the proposed research to the priorities and needs of the NHS, and does it offer a health/healthcare solution with demonstrable benefit to patients?
b) Does the application demonstrate an awareness and understanding of previous relevant research or developments in this area?
c) To what extent does the proposed research add distinct value or provide an advance to what is already known from other work currently in progress or already completed in this area?

An economic evalaution of this kind has not been done before and hence is of benefit to the NHS and patients.

2. QUALITY of the proposed work

2a. Research design

a) Is the proposed research of high quality and does it address the stated objectives?
b) How convincing and coherent is the proposed rationale and approach?
c) Is the proposed design and methodology for all elements of the research well defined, appropriate, valid, robust and feasible within the timeframe and resources requested?
d) What are the strengths and weaknesses of the research design as proposed?

The economic evaluation is well described and is of high quality. The methods are standard and are explained in sufficient detail to show that the team have expertise in this area and have considered all aspects carefully, including both the NHS and societal perspective.
The costs will be collected using the CSRI, a valid instrument in mental health. Have the applicants also considered the RUD (Resource Use in Dementia), either as a check or to provide further information? This is widely used in International trials.

2. QUALITY of the proposed work

2b. Work plan and proposed management arrangements

a) Do the work plan and project management arrangements give confidence that proposed milestones will be met within the specified timeframe?
b) Can you identify any difficulties that the applicants are likely to encounter in meeting their milestones?
c) Have the major ethical, scientific, technical and organisational challenges, as well as any issues around intellectual property rights, been identified and will they be addressed adequately?
d) Are the necessary clinical, academic or organisational links needed to support the research, or help translate it into practice, in place?
e) Is long-term, large scale programme grant funding appropriate; is there added value over and above the dividends from the individual elements?

The main issues will be recruitment to the trial and collection of all relevant costs. Will the applicants do an interim economic evaluation or a check on the data, to ensure all the relevant data are being collected?

---

3. STRENGTH of the research team

a) Are the applicants familiar with the methodologies outlined in their application and are they well qualified to undertake the proposed work on the basis of track record in relevant areas (as judged by publication output and previous research funding)?
b) Does the research team have the necessary breadth and depth of expertise to deliver the planned work?
c) Are the roles of the team members clearly described, and is the overall team well coordinated?
d) If the lead applicant is inexperienced, does he/she have appropriate support (e.g. from their host institution and more senior colleagues) to deliver the work plan?

The team show relevant experience and expertise and have carried out economic evaluation on other data previously and have published widely.

---

4. IMPACT of the proposed work

a) Have the applicants outlined an appropriate and adequate approach to disseminating the result of their research (including engaging with healthcare planners and/or policy makers)? Could this be developed further?
b) Considering the plans for dissemination, what is the likelihood of a significant benefit to the NHS and patients within 3-5 years of the end of the funding period?
c) Have the applicants identified any new intellectual property that will be produced during the course of this research and are there plans to protect and exploit it?

The cost effectiveness part of this proposal is essential for the overall assessment of the toolkit.

---

5. VALUE for money (justification for proposed costs)

a) Are the resources required for this research, including staffing, clearly justified? Are they essential for the work proposed?
b) Where the application offers arguments about financial benefits, please assess how realistic these might be.
c) Taking into account the expected benefits of the work proposed and the level of resources requested, does this application represent good value for money?
d) If required, are funds requested for Support and Treatment Costs appropriate?

The proposal indicates good value for money for the economic evaluation.

---

6. INVOLVEMENT of patients and the public

Where applicable (N.B. if there is no patient and public involvement, please address question d below)

a) What is your assessment of the patient and public involvement (if any) in the development of the application including involvement in: identifying the research topic; prioritising the research questions; preparing the application (e.g. contributing to the research design)?

b) What is your assessment of any proposed plans for patient and public involvement throughout the duration of the research? Can you identify particular strengths, weaknesses and/or areas for improvement?
c) Are the resources set aside for patient and public involvement appropriate - including plans for a training and support budget?
d) If there is no patient and public involvement in the application, what is your assessment of the reasons given for this?

n/a

7. Additional comments

Do you have any other comments or suggestions for how the proposed research might be improved? If so, please indicate whether you see these as critical factors.

PGfAR Competition 13 Stage 2

Peer Review Form

**Application Reference**	RP-PG-0612-20004
**Application Title**	Achieving Quality and Effectiveness in Dementia Using Crisis Teams (AQUEDUCT)
**Applicant**	Professor Martin Orrell
**Applicant Organisation**	University College London
**Total Amount Requested**	£1,978,647.00
**Reviewer Number**	4

**Reviewer Expertise**	Researcher in a broadly related field<br>end of life care, old age, dementia, bereavement

1. RELEVANCE of the proposed research

a) How relevant and important is the proposed research to the priorities and needs of the NHS, and does it offer a health/healthcare solution with demonstrable benefit to patients?
b) Does the application demonstrate an awareness and understanding of previous relevant research or developments in this area?
c) To what extent does the proposed research add distinct value or provide an advance to what is already known from other work currently in progress or already completed in this area?

Maintaining care at home and reducing hospital admissions for people with dementia is a national NHS priority. The study has potential to provide services that will facilitate and maintain care of people with dementia at home at times of crisis. There is good understanding of previous relevant research and development in the area of care provision.

2. QUALITY of the proposed work

2a. Research design

a) Is the proposed research of high quality and does it address the stated objectives?
b) How convincing and coherent is the proposed rationale and approach?
c) Is the proposed design and methodology for all elements of the research well defined, appropriate, valid, robust and feasible within the timeframe and resources requested?
d) What are the strengths and weaknesses of the research design as proposed?

The description of the study demonstrates that high quality and rigorous research will be conducted and the team have addressed concerns raised in the first round.
A feasibility study is proposed the outcome of which should determine whether to proceed to an RCT (WP3).
The RCT plan is supported by high level expertise in the methodology however I have reservations regarding possible 'contamination', such as the skills and expertise of individual service providers on the DCTs and existing protocols used by different teams plus the diversity and complexity of crisis situations influencing outcomes.

2. QUALITY of the proposed work

2b. Work plan and proposed management arrangements

a) Do the work plan and project management arrangements give confidence that proposed milestones will be met within the specified timeframe?
b) Can you identify any difficulties that the applicants are likely to encounter in meeting their milestones?
c) Have the major ethical, scientific, technical and organisational challenges, as well as any issues around intellectual property rights, been identified and will they be addressed adequately?
d) Are the necessary clinical, academic or organisational links needed to support the research, or help translate it into practice, in place?
e) Is long-term, large scale programme grant funding appropriate; is there added value over and above the dividends from the individual elements?

Concerns expressed in the first round have been addressed by the research team.
Until feasibility is determined I do not believe funding for an RCT is appropriate.

---

3. STRENGTH of the research team

a) Are the applicants familiar with the methodologies outlined in their application and are they well qualified to undertake the proposed work on the basis of track record in relevant areas (as judged by publication output and previous research funding)?
b) Does the research team have the necessary breadth and depth of expertise to deliver the planned work?
c) Are the roles of the team members clearly described, and is the overall team well coordinated?
d) If the lead applicant is inexperienced, does he/she have appropriate support (e.g. from their host institution and more senior colleagues) to deliver the work plan?

There is very good evidence that the team is highly experienced in conducting a research of this scale and design.

---

4. IMPACT of the proposed work

a) Have the applicants outlined an appropriate and adequate approach to disseminating the result of their research (including engaging with healthcare planners and/or policy makers)? Could this be developed further?
b) Considering the plans for dissemination, what is the likelihood of a significant benefit to the NHS and patients within 3-5 years of the end of the funding period?
c) Have the applicants identified any new intellectual property that will be produced during the course of this research and are there plans to protect and exploit it?

A comprehensive dissemination plan is provided.
The outcomes will have significant benefit to the NHS and patients.
New intellectual property will be produced - this has been addressed adequately.

---

5. VALUE for money (justification for proposed costs)

a) Are the resources required for this research, including staffing, clearly justified? Are they essential for the work proposed?
b) Where the application offers arguments about financial benefits, please assess how realistic these might be.
c) Taking into account the expected benefits of the work proposed and the level of resources requested, does this application represent good value for money?
d) If required, are funds requested for Support and Treatment Costs appropriate?

Not convinced that there is value for money for WP3 (RCT). Support funding for a feasibility study to determine necessity and appropriateness of RCT.

---

6. INVOLVEMENT of patients and the public

Where applicable (N.B. if there is no patient and public involvement, please address question d below)
a) What is your assessment of the patient and public involvement (if any) in the development of the application including involvement in: identifying the research topic; prioritising the research questions; preparing the application (e.g. contributing to the research design)?
b) What is your assessment of any proposed plans for patient and public involvement throughout the duration of the research? Can you identify particular strengths, weaknesses and/or areas for improvement?
c) Are the resources set aside for patient and public involvement appropriate - including plans for a training and support budget?
d) If there is no patient and public involvement in the application, what is your assessment of the reasons given for this?

Increased involvement of consumers has been strengthened - based on proposal development following first round review.

---

7. Additional comments

Do you have any other comments or suggestions for how the proposed research might be improved? If so, please indicate whether you see these as critical factors.

I believe it is critical that feasibility be established before proceeding to RCT, consequently I would recommend that funding for RCT be applied for based on the outcome of the feasibility study.

National Institute for Health Research

PGfAR Competition 13 Stage 2

Lay Review Form

**Application Reference**	RP-PG-0612-20004
**Application Title**	Achieving Quality and Effectiveness in Dementia Using Crisis Teams (AQUEDUCT)
**Applicant**	Professor Martin Orrell
**Applicant Organisation**	University College London
**Total Amount Requested**	£1,978,647.00
**Reviewer Number**	5

**Reviewer Expertise**	Member of public with a more general view<br>Cardiology and Urological Services and also Mental Health Services. Social Care: Diabetes; COPD; Parkinsons Disease; Dementia (carer roles)

1. RELEVANCE of the proposed research

a) How relevant and important is the proposed research to the priorities and needs of the NHS, and does it offer a health/healthcare solution with demonstrable benefit to patients?
b) Does the application demonstrate an awareness and understanding of previous relevant research or developments in this area?
c) To what extent does the proposed research add distinct value or provide an advance to what is already known from other work currently in progress or already completed in this area?

The relevance of the proposed research has impact in addressing an area of need relating to the provision of informal care and crisis service for those with Dementia. The priority is important in the context of the NHS and there is a need which is clearly stated to enhance the knowledge base in managing crisis for this client group and wider social networks. There is demonstrable benefit in the proposal in developing the knowledge base regarding the impact of such a crisis intervention. There is further clarity required in how the impact of the applied research proposal is measured in terms of outcomes for the individuals. There is an awareness and understanding of previous research and the need for the development of the knowledge base around the subject. The proposed research adds value in addressing the need for academic knowledge around the use of crisis teams in Dementia care, but it is not clear how the development of the Resource Kit can be fully applied in relation to wider team structures within the NHS.

2. QUALITY of the proposed work

2a. Research design

a) Is the proposed research of high quality and does it address the stated objectives?
b) How convincing and coherent is the proposed rationale and approach?
c) Is the proposed design and methodology for all elements of the research well defined, appropriate, valid, robust and feasible within the timeframe and resources requested?
d) What are the strengths and weaknesses of the research design as proposed?

The proposed research is detailed and addresses the objectives in providing a study which relates to the application of the Resource Kit relating to crisis intervention. The proposed rationale is of interest and has considered the ethical consideration of informed consent and participation. There is further consideration required relating to how the different conditions within the Dementia diagnosis influence the practicalities of management of crisis. This can be seen in how there is a need for further detail relating to how outcomes are evaluated on how the Resource Kit and the subsequent qualitative work considers participants and their wider networks.

There is a need in the qualitative study to further develop answers to how the participants will be aware of the mechanisms of the implementation of the intervention and how this is considered in making informed answers. The proposed research discusses that it is not known if AQUADECT will reduce hospital admissions for those with Dementia, but it is hoped that the Resource Kit will be adopted nationally as best practice. There requires further clarity as to the direct impact of the Resource Kit on such outcomes and indeed on the quality of life for those within crisis and their wider networks. The proposed rationale is based mainly on a measurement that considers admission within a hospital setting as being a key marker of the escalation of a crisis. There is further consideration that these admissions cannot be fully used as an objective measurement of the direct impact of the Resource Kit, as there are further variables related to socio-economic demography and resources for admission. There are strengths in the use of clear ethical consideration to the involvement of participants and the use of data. The weaknesses relating to the research design are based on the lack of applied roles within PPI and the lack of definitive measurement of the impact of the Resource Kit.

2. QUALITY of the proposed work

2b. Work plan and proposed management arrangements

a) Do the work plan and project management arrangements give confidence that proposed milestones will be met within the specified timeframe?
b) Can you identify any difficulties that the applicants are likely to encounter in meeting their milestones?
c) Have the major ethical, scientific, technical and organisational challenges, as well as any issues around intellectual property rights, been identified and will they be addressed adequately?
d) Are the necessary clinical, academic or organisational links needed to support the research, or help translate it into practice, in place?
e) Is long-term, large scale programme grant funding appropriate; is there added value over and above the dividends from the individual elements?

The work plan and project management arrangements are suitable regarding the implementation of the specified timeframe. There are no obvious challenges relating to the meeting of the milestones, but there is ethical consideration in how the informed perspectives are developed relating to the qualitative aspect of the proposal. In order for the qualitative focus group participants to consider the impact of the work, the need to be informed about the nature of the intervention. This creates a duality in how their perspectives objectively consider the change of management of crisis and not the Resource Kit itself. There are issues relating to the applied use of the intellectual property of the Resource Kit and its assertion of use as best practice considering the limited measurements used. There are the necessary clinical and academic links to support the development of the research proposal and there is a need for greater knowledge to a suitable extent based on the crisis experience of individuals with Dementia and their networks.

3. STRENGTH of the research team

a) Are the applicants familiar with the methodologies outlined in their application and are they well qualified to undertake the proposed work on the basis of track record in relevant areas (as judged by publication output and previous research funding)?
b) Does the research team have the necessary breadth and depth of expertise to deliver the planned work?
c) Are the roles of the team members clearly described, and is the overall team well coordinated?
d) If the lead applicant is inexperienced, does he/she have appropriate support (e.g. from their host institution and more senior colleagues) to deliver the work plan?

The applicants stated within the research proposal are familiar with the methodologies related to the application and are well qualified to undertake the work on the basis of their track records. There is

particular strength within the field of working with people and families related to the Dementia diagnosis and the wider networks related to the provision of care. The roles of the team members are clearly described, but there requires further clarity of the role of the PPI within the proposal and within the context of how such knowledge is cascade as intellectual property. The lead applicant has relevant experience and skills related to the ongoing management of the proposal and the implementation of the timescales.

---

4. IMPACT of the proposed work

a) Have the applicants outlined an appropriate and adequate approach to disseminating the result of their research (including engaging with healthcare planners and/or policy makers)? Could this be developed further?
b) Considering the plans for dissemination, what is the likelihood of a significant benefit to the NHS and patients within 3-5 years of the end of the funding period?
c) Have the applicants identified any new intellectual property that will be produced during the course of this research and are there plans to protect and exploit it?

The proposed work has an adequate approach to the dissemination of the findings, but there is further evidence which is required relating to the significant benefit to the patients. The main focus of the benefit is through the development of the intellectual property and the Resource Kit and how such materials clearly will impact on care delivery is not clearly seen. This is considered further in how the use of standard materials will ensure service delivery which is variable in area to area in terms of demography and organisational structure. There is new intellectual property gained and the plans to use this are central to the research proposal in the form of the developed Resource Kit.

---

5. VALUE for money (justification for proposed costs)

a) Are the resources required for this research, including staffing, clearly justified? Are they essential for the work proposed?
b) Where the application offers arguments about financial benefits, please assess how realistic these might be.
c) Taking into account the expected benefits of the work proposed and the level of resources requested, does this application represent good value for money?
d) If required, are funds requested for Support and Treatment Costs appropriate?

The resources for the research are clear to an extent, but require further clarity in certain areas such as those allocated to involvement. There is an interesting point where money is attributed to the process of consenting for participation on Page 75 and a time allocated to this process of 30 minutes. It is questioned how this actively takes place, when to make an informed decision, additional time and parameters may be necessary to make a choice based on capacity. The benefits of the work are not fully clear as the reduction in admissions to hospital are not guaranteed and the impact on quality of life cannot be fully evaluated in relation to differences in service provision.

---

6. INVOLVEMENT of patients and the public

Where applicable (N.B. if there is no patient and public involvement, please address question d below)
a) What is your assessment of the patient and public involvement (if any) in the development of the application including involvement in: identifying the research topic; prioritising the research questions; preparing the application (e.g. contributing to the research design)?
b) What is your assessment of any proposed plans for patient and public involvement throughout the duration of the research? Can you identify particular strengths, weaknesses and/or areas for improvement?
c) Are the resources set aside for patient and public involvement appropriate - including plans for a training and support budget?
d) If there is no patient and public involvement in the application, what is your assessment of the reasons given for this?

The patient and public involvement within the proposal is consistent and there is involvement of the wider public within the initial stages. There is involvement in the form of a Management Group for the research proposal and a Reference Group which comprises of representatives of the wider patient and public. There is further information which is required and to how these will be recruited

and also how the intellectual property ownership reflects the experience of those who have participate from a PPI perspective. The costings for PPI within the research proposal are unclear in how what work is carried out within the allocated subcontracts and how this work will be evidenced as well.

---

7. Additional comments

Do you have any other comments or suggestions for how the proposed research might be improved? If so, please indicate whether you see these as critical factors.

The proposed research has a good ethos, but there is a lack of clarity as to how the outcomes of quality of life will be effectively measured and value for money from a patient and public perspective gained. The proposed research could demonstrate greater correlation of the evidence base rather than then singular development of the tool of intellectual property in the form of the Resource Kit.

National Institute for Health Research

PGfAR Competition 13 Stage 2

Peer Review Form

**Application Reference**	RP-PG-0612-20004
**Application Title**	Achieving Quality and Effectiveness in Dementia Using Crisis Teams (AQUEDUCT)
**Applicant**	Professor Martin Orrell
**Applicant Organisation**	University College London
**Total Amount Requested**	£1,978,647.00
**Reviewer Number**	6

**Reviewer Expertise**	Researcher in the same/a very similar field<br>Methodologist<br>Dr Romeo's main focus is on economic analyses and economic evaluations of interventions across in mental health.

1. RELEVANCE of the proposed research

a) How relevant and important is the proposed research to the priorities and needs of the NHS, and does it offer a health/healthcare solution with demonstrable benefit to patients?
b) Does the application demonstrate an awareness and understanding of previous relevant research or developments in this area?
c) To what extent does the proposed research add distinct value or provide an advance to what is already known from other work currently in progress or already completed in this area?

Crises in individuals with dementia are peculiar to the individual and come in all kinds of formats. In that case, every crisis has a unique solution. This can make it very difficult to prevent or plan for all crises even if there is a willingness to create a strategic plan. There has been research conducted by Vroomen et al (2013)* which explore an operational framework to compile types of crisis stressors and recommendations from the crisis.
The applicants rightly indicate that crisis teams have been developed to prevent avoidable hospital admissions and support people in their homes. While there are no accepted standards of good practice, there has been research to suggest that crisis teams for older people with mental health needs reduce hospital admission rates. It is there unclear what would be the added utility of the proposed programme of research.

*Vroomen, Janet MacNeil, et al. "Reviewing the definition of crisis in dementia care." BMC geriatrics 13.1 (2013): 10.

2. QUALITY of the proposed work

2a. Research design

a) Is the proposed research of high quality and does it address the stated objectives?
b) How convincing and coherent is the proposed rationale and approach?
c) Is the proposed design and methodology for all elements of the research well defined,

appropriate, valid, robust and feasible within the timeframe and resources requested?
d) What are the strengths and weaknesses of the research design as proposed?

The proposed research was presented clearly and to a large extent addresses the stated objectives. Where I am less convinced is in terms of the measure of outcome chosen and following on from that the economic evaluation of the Resource Kit (RK). The economic evaluation of the RK does not seem to mesh well with the rest of the application. Perhaps the applications should have considered focusing on emergency hospitalisations avoided as an outcome measure. Although it was suggested by NIHR to look at number of admissions, it seems to me that this measure could be misleading as it could be that an individual with dementia in old age may - quite validly – need to be treated with medical inputs which can only be provided in a hospital setting. There will however be crises which could be dealt with at home or in a less intensive setting (than a hospital) and it is that unnecessary admission which should be the focus. It may seem that the goal post is being changed but am not convinced that the primary outcome is a useful one.
It was suggested that aim of the economic analysis would be to estimate the lifetime incremental cost per quality-adjusted life year gained from the RK versus treatment as usual (TAU). I am in doubt why the team has chosen to focus on QALYs and not on the primary outcome to be assessed by the programme – perhaps as well as QALYs? It seems to me that commissioners of services would be keen to assess whether the introduction of such an intervention will not only improve the well-being (in reducing unnecessary admissions) of its service users but also bring about reductions in service use (and costs) in high cost areas such as hospitalisations (which could have been avoided).
The applicants will be using DEMQOL-U and DEMQOL-Proxy measures to explore QALY gains, however more recent evidence into the validity and application of the these two measures by Mulhern et al (2013) suggest that these measures should be used alongside measures such as EQ-5D. While there is some discussion on using the EQ-5D-5L it is unclear where this instrument would sit with the evaluations. This needs to be justified and made clearer.

The applicants will also extrapolate costs and outcomes beyond the end of the trial. While this is interesting academically, this is an aged population and it is unclear how preventing a crisis could potentially extend life. Justification is needed. Further, modelling can be quite involved and built on (potentially a large number of) assumptions especially where the data are not available - which by the applicants admission might be the case. It is unclear what would be the added utility of such a model.

---

2. QUALITY of the proposed work

2b. Work plan and proposed management arrangements

a) Do the work plan and project management arrangements give confidence that proposed milestones will be met within the specified timeframe?
b) Can you identify any difficulties that the applicants are likely to encounter in meeting their milestones?
c) Have the major ethical, scientific, technical and organisational challenges, as well as any issues around intellectual property rights, been identified and will they be addressed adequately?
d) Are the necessary clinical, academic or organisational links needed to support the research, or help translate it into practice, in place?
e) Is long-term, large scale programme grant funding appropriate; is there added value over and above the dividends from the individual elements?

Concern is expressed about the inclusion of long-term modelling for the evaluation in addition to the primary analysis for the evaluation.
The difficulties the applicants are likely to encounter relate to my concerns above about data quality for the model. The funding seems appropriate in most areas. However, I am very concerned about the allocations to the Health Economist.

---

3. STRENGTH of the research team

a) Are the applicants familiar with the methodologies outlined in their application and are they well qualified to undertake the proposed work on the basis of track record in relevant areas (as judged by publication output and previous research funding)?
b) Does the research team have the necessary breadth and depth of expertise to deliver the planned work?
c) Are the roles of the team members clearly described, and is the overall team well coordinated?

d) If the lead applicant is inexperienced, does he/she have appropriate support (e.g. from their host institution and more senior colleagues) to deliver the work plan?

Roles are clearly defined and the team strong academcially. Their individual breadth of experience is impressive

---

4. IMPACT of the proposed work

a) Have the applicants outlined an appropriate and adequate approach to disseminating the result of their research (including engaging with healthcare planners and/or policy makers)? Could this be developed further?
b) Considering the plans for dissemination, what is the likelihood of a significant benefit to the NHS and patients within 3-5 years of the end of the funding period?
c) Have the applicants identified any new intellectual property that will be produced during the course of this research and are there plans to protect and exploit it?

The applicants have outlined an appropriate and adequate approach to disseminating the result of their research. However, I am unsure of what would be th measurable benefit to the NHS from this programme.

---

5. VALUE for money (justification for proposed costs)

a) Are the resources required for this research, including staffing, clearly justified? Are they essential for the work proposed?
b) Where the application offers arguments about financial benefits, please assess how realistic these might be.
c) Taking into account the expected benefits of the work proposed and the level of resources requested, does this application represent good value for money?
d) If required, are funds requested for Support and Treatment Costs appropriate?

The health economics funding seems inadequate for the work involved.

---

6. INVOLVEMENT of patients and the public

Where applicable (N.B. if there is no patient and public involvement, please address question d below)
a) What is your assessment of the patient and public involvement (if any) in the development of the application including involvement in: identifying the research topic; prioritising the research questions; preparing the application (e.g. contributing to the research design)?
b) What is your assessment of any proposed plans for patient and public involvement throughout the duration of the research? Can you identify particular strengths, weaknesses and/or areas for improvement?
c) Are the resources set aside for patient and public involvement appropriate - including plans for a training and support budget?
d) If there is no patient and public involvement in the application, what is your assessment of the reasons given for this?

Patient involvement in the application seems acceptable

---

7. Additional comments

Do you have any other comments or suggestions for how the proposed research might be improved? If so, please indicate whether you see these as critical factors.

The programme does not read as a cohesive programme grant. Indeed, the economic evaluation seems disjointed from the rest of the application. Further thought should be given on the primary measure of outcome. It is unclear whether the research can shown whether the intervention can bring about significant improvements for the patients such as unceccessary hospital admission are evidenced.

PGfAR Competition 13 Stage 2

Peer Review Form

**Application Reference**	RP-PG-0612-20004
**Application Title**	Achieving Quality and Effectiveness in Dementia Using Crisis Teams (AQUEDUCT)
**Applicant**	Professor Martin Orrell
**Applicant Organisation**	University College London
**Total Amount Requested**	£1,978,647.00
**Reviewer Number**	7

**Reviewer Expertise**	Researcher in the same/a very similar field<br>sleep in dementia and caring, neuropsychology, gerontology

1. RELEVANCE of the proposed research

a) How relevant and important is the proposed research to the priorities and needs of the NHS, and does it offer a health/healthcare solution with demonstrable benefit to patients?
b) Does the application demonstrate an awareness and understanding of previous relevant research or developments in this area?
c) To what extent does the proposed research add distinct value or provide an advance to what is already known from other work currently in progress or already completed in this area?

a) The proposed research has both relevance and importance for the NHS and for patients. There are clear benefits for patients within the proposed programme of work, and the outcomes, if efficacious, will inevitably deliver significant savings for the NHS both in terms of care burden and financial cost.
b) The application is presented in a highly cogent way, with reference to relevant previous research published in well respected sources; conducted by independent researchers; and also by members of the study team, whose programmatic work coalesces in this submission and adds significant strength to it.
c) As above, the proposed programme of work follows naturally from previous research conducted in this field and is significantly informed by previous programmatic work conducted by various combinations of the study team members at different time-points over the last few years.

2. QUALITY of the proposed work

2a. Research design

a) Is the proposed research of high quality and does it address the stated objectives?
b) How convincing and coherent is the proposed rationale and approach?
c) Is the proposed design and methodology for all elements of the research well defined, appropriate, valid, robust and feasible within the timeframe and resources requested?
d) What are the strengths and weaknesses of the research design as proposed?

a) The proposed programme of study is of very high quality and, should it proceed to the stated

plans, is highly likely to satisfactorily address the stated objectives.
b) The natural extension of prior work conducted by various members of the study team to inform this programme of work makes this proposal highly convincing and coherent. There were a few minor issues (elaborated upon below), but there was nothing of any significant concern in the presented proposal that would likely jeopardise a fruitful and beneficial line of enquiry.
c) The proposed methods for the three work packages are well justified and defined, they dovetail together well and are appropriately designed to meet the stated objectives of this programme of work. The proposal utilises valid and robust measures and approaches (e.g. to ethical, financial, feasibility, managerial and power considerations / constraints). There is a good likelihood that this programme of work can be successfully delivered to the timescales and budgets described.
d) The strengths of the study stem from: 1) the strength, experience, diverse range (yet cohesive nature) of the research team, many of whom have successfully collaborated on a number of large studies in the past; 2) as a result of 1), the study team are very well positioned to have a very clear understanding of the current scientific and political issues abound at the present time in order to design a programme of work that has both pertinence and utility; 3) the natural follow-on of each work package from previous ones and from prior successfully delivered and published work; and 4) the urgent need for cohesive service provision for older people with dementia in community settings set against the backdrop of an ageing population and increasing prevalence of dementia makes this proposal timely, important and in line with prioritised government policy.
Weaknesses of the study are minimal, but do include:
1) a potential ethical issue in WP3 around a proportion of the DCT control groups receiving the Resource Kit. It was unclear in the proposal what proportion of control DCTs would receive this, how these DCTs would be identified; and a potential ethical conflict for those control DCTs not given access to the Resource Kit. Might they be able to access this after the conclusion of their involvement in the study?
2) Page 21 line 10: discusses the potential for studies to gain ethical approval and collect data successfully despite an 'absence of baseline measures' and cites Johnson (2005) who is a member of the study team. This raises the potential issue surrounding anticipated difficulties with gaining ethical approval for such work. If this has been an issue in the past what is the likelihood that this may occur again, what strategies might be employed to minimise these, and does previous work allow for a change in approach to NRES that might facilitate timely ethical approval and permit the programme to proceed according to timescale and budget?
3) Page 29: Field testing the Implementation Measure: the proposal discusses sample sizes of 15 DCTs and 10 people with dementia, however it was unclear in the proposal how these sample sizes were determined. It was also unclear how the study team intended to 'assess the internal structure' or conduct 'item analysis' on this measure. exploratory / confirmatory factor analysis for eg.?
4) Page 30: The 'process mapping tool' and 'feasibility screening checklist' (also in WP3 on page 31) are poorly defined, it is unclear what will constitute these elements, or how they will be applied and the data they generate interpreted.
5)The low to moderate implementation measure score as a threshold to inclusion is mentioned on page 31 in WP3. It is unclear what this threshold might be, or whether this has yet been defined. If it has not yet been defined then at what stage will it be, how will this definition be made and by whom?
6) Page 32: it is unclear how an estimation of blinding integrity will be carried out and by whom.
7) Page 33: This is perhaps the moist major issue identified: The study team hypothesise a reduction from 70% to 50% of admissions with the use of the resource kit. This reduction of 0.2 then informs an elegant and well presented power calculation and sample size justification. However, it is unclear how the study team have estimated that a 20% reduction is expected or realistic. A stronger justification for this expectation is required to then have fuller confidence in the subsequent power calculation.
8) Page 34: A 'de novo cost effectiveness model' is proposed; The query here is how realistic is it to assume a veracious outcome to this model if it is attempting to make lifetime time horizon predictions based on data from a 26 week study. Are there any existing models that have been developed in a similar vein to add support to such an endeavour?
9) Page 44: There was no existing IP search, but the experience of the study team would certainly mean that they would be well aware of any existing IP, so no concerns.

---

2. QUALITY of the proposed work

2b. Work plan and proposed management arrangements

a) Do the work plan and project management arrangements give confidence that proposed milestones will be met within the specified timeframe?
b) Can you identify any difficulties that the applicants are likely to encounter in meeting their milestones?

c) Have the major ethical, scientific, technical and organisational challenges, as well as any issues around intellectual property rights, been identified and will they be addressed adequately?
d) Are the necessary clinical, academic or organisational links needed to support the research, or help translate it into practice, in place?
e) Is long-term, large scale programme grant funding appropriate; is there added value over and above the dividends from the individual elements?

---

a) These are well presented and there are no concerns that the proposed milestones should not be met in a timely fashion
b) There has been a well presented set of contingencies to cover for any potential difficulties in progression, so no concerns here.
c) These have been well thought through and appropriately discussed in sufficient detail in the proposal, so no added concerns here.
d) There is clear and evident support from well respected establishments within academia and the NHS to capably support this programme of work, no concerns.
e) The cohesive nature of this programme's three work packages which again flow from previous funded and published work justifies such a grant.

---

3. STRENGTH of the research team

a) Are the applicants familiar with the methodologies outlined in their application and are they well qualified to undertake the proposed work on the basis of track record in relevant areas (as judged by publication output and previous research funding)?
b) Does the research team have the necessary breadth and depth of expertise to deliver the planned work?
c) Are the roles of the team members clearly described, and is the overall team well coordinated?
d) If the lead applicant is inexperienced, does he/she have appropriate support (e.g. from their host institution and more senior colleagues) to deliver the work plan?

---

a - d:
As stated above the characteristics of the research team is a particular strength of this application. They are all very highly qualified and experienced. They have received multiple grants totalling many millions of pounds to conduct research that has had a seminal impact on the field. They have collaborated in various combinations over many studies across many years. There is no doubt that this research group are entirely well placed to conduct this programme of research to its planned conclusions, to time and to budget.

---

4. IMPACT of the proposed work

a) Have the applicants outlined an appropriate and adequate approach to disseminating the result of their research (including engaging with healthcare planners and/or policy makers)? Could this be developed further?
b) Considering the plans for dissemination, what is the likelihood of a significant benefit to the NHS and patients within 3-5 years of the end of the funding period?
c) Have the applicants identified any new intellectual property that will be produced during the course of this research and are there plans to protect and exploit it?

---

a) There is a clear dissemination plan and several team members are closely linked with policy makers.
b) There is a high likelihood of benefits to the NHS and to patients within 3 - 5 years from the conclusion of this study and these have been satisfactorily reported and described in the proposal.
c) There is a high likelihood of the generation of new IP in the form of the Implementation Measure, the Resource Kit and the Home Treatment Protocol. Some members of the team are experienced in the generation and management of exploitation of new IP and plans are described well in the proposal to this end.

---

5. VALUE for money (justification for proposed costs)

a) Are the resources required for this research, including staffing, clearly justified? Are they essential for the work proposed?
b) Where the application offers arguments about financial benefits, please assess how realistic these might be.
c) Taking into account the expected benefits of the work proposed and the level of resources

requested, does this application represent good value for money?
d) If required, are funds requested for Support and Treatment Costs appropriate?

---

a) There is a good justification for the resources required for this programme of work, staffing is realistically described and so there are no concerns regarding resource requirements.
b) There is an estimation of potential financial benefits to the NHS, obviously these are dependent on the successful delivery of this programme of work and its subsequent influence on policy with respect to dementia care in this country. Should the programme be executed successfully and influence policy as predicted then there is little doubt that there will be significant cost-savings to the NHS in both direct costs and the more elusive hidden costs associated with hospital admissions of this particularly vulnerable cohort. These savings may well be in line with the £59 million per year described in the proposal, they may be significantly higher than this.
c) as above - yes.
d) These are not substantial and their justification has been well argued.

---

6. INVOLVEMENT of patients and the public

Where applicable (N.B. if there is no patient and public involvement, please address question d below)
a) What is your assessment of the patient and public involvement (if any) in the development of the application including involvement in: identifying the research topic; prioritising the research questions; preparing the application (e.g. contributing to the research design)?
b) What is your assessment of any proposed plans for patient and public involvement throughout the duration of the research? Can you identify particular strengths, weaknesses and/or areas for improvement?
c) Are the resources set aside for patient and public involvement appropriate - including plans for a training and support budget?
d) If there is no patient and public involvement in the application, what is your assessment of the reasons given for this?

---

a) and b) Patient and Public Involvement has been well utilised in the development of this programme of work with a representative involved as a co-applicant. The recruitment and retention of family carers (or ex-carers) is planned throughout the duration of this programme of work and their involvement is integral to the planned work.
c) There is sufficient funding requested within this proposal to support and retain these PPI members.
d) N/A.

---

7. Additional comments

Do you have any other comments or suggestions for how the proposed research might be improved? If so, please indicate whether you see these as critical factors.

None.

PGfAR Competition 13 Stage 2

Peer Review Form

**Application Reference**	RP-PG-0612-20004
**Application Title**	Achieving Quality and Effectiveness in Dementia Using Crisis Teams (AQUEDUCT)
**Applicant**	Professor Martin Orrell
**Applicant Organisation**	University College London
**Total Amount Requested**	£1,978,647.00
**Reviewer Number**	8

**Reviewer Expertise**	Researcher in a broadly related field<br>older people, long-term conditions, dementia care, qualitative research, participatory approaches and action research, user involvement, practice and service development

1. RELEVANCE of the proposed research

a) How relevant and important is the proposed research to the priorities and needs of the NHS, and does it offer a health/healthcare solution with demonstrable benefit to patients?
b) Does the application demonstrate an awareness and understanding of previous relevant research or developments in this area?
c) To what extent does the proposed research add distinct value or provide an advance to what is already known from other work currently in progress or already completed in this area?

This proposal addresses the need to establish the effectiveness of crisis teams in preventing admission to hospital. This is an important issue of course. There are many points at which admission to hospital may be averted, and this proposed research focusses on those already in distress in the community and where admission is near-inevitable without further intervention. Not acknowledged in the proposal, are wider health and social care developments that seek to avert crises taking place at a much earlier stage. However, there is at present, a need for intervention at the point of crises and there is to date little systematic evaluation of such services so this proposed research has the potential to make a useful contribution.

2. QUALITY of the proposed work

2a. Research design

a) Is the proposed research of high quality and does it address the stated objectives?
b) How convincing and coherent is the proposed rationale and approach?
c) Is the proposed design and methodology for all elements of the research well defined, appropriate, valid, robust and feasible within the timeframe and resources requested?
d) What are the strengths and weaknesses of the research design as proposed?

Initial stages of the work are designed to further develop the intervention (Home Treatment Protocol) and take it to a cluster RCT and implement in routine practice. It is worth noting that there

has already been significant work on developing the intervention so it is not fully clear what additional benefit there will be from the initial stages of the research.

There are a few areas of inconsistency and other areas require further elaboration e.g.

- aims and objectives in section 9 are inconsistently stated.

- the applicants claim that it is feasible to recruit to the trail at the point of crises but this will be ethically and practically very challenging and the applicants rather understate the challenge here.

- I could not ascertain whether individual services will be randomised or Trusts (which could have 2 or 3 services) - either way, ensuring that there is no cross-over learning between teams will need to be addressed (and is not at the moment).

- as in my comment above, this is end-stage crisis intervention and there are many other services already that seek to prevent crises. Therefore the individual composition of health and social care services within an area is critical to the nature and outcomes of intervention from a specific crisis intervention team. This is not reflected in the proposal, and requires at the least an account of local service patterns and a view of party of service context in which the intervention services are working.

- no rationale is provided for the selection of secondary outcomes. The outcomes for the person with dementia appear to be being collected at 12 and 26 weeks post-intervention but not at baseline. Usefully, there are also outcomes for the carer but I would recommend that this needs to expand to include, for example, any health incident of the carer and social network information. We know that admission to hospital is very often prompted by 'carer breakdown', perhaps because they have a period of being physically unwell or a family stressor event such as a bereavement. This aspect requires much more attention in the proposal which seems to be more predicated on it being changes in the person with the diagnosis that precipitates admission.

- there is a need to better define and operationalize 'crisis' - the argument on page 33 that admissions in areas with a crisis intervention team are 70% of the anticipated admission rate are unable to be claimed in the absence of a better articulation of 'crisis' and the case here that teams reduce admissions by 30% is very weak.

- the attrition rate is likely to be greatly underestimated. It has been modelled on the 15% attrition of the REMCARE study but these are vastly different samples, REMCARE working with people who are not in crisis.

- there is a small qualitative element to the proposed research which will interview 6 staff and 6 carers in teams where it has been 'easier to implement' the protocol and 6 where it has been harder - however, what is the rationale for this number of interviews? and crucially, how will ease of implementation be judged in order to inform the stated selection process?

- there is passing mention of the 'ethnographic analysis of observation field notes', however, there is no indication elsewhere that such data is to be collected, where and from whom, so this seems to be a rather errant statement.

---

2. QUALITY of the proposed work

2b. Work plan and proposed management arrangements

a) Do the work plan and project management arrangements give confidence that proposed milestones will be met within the specified timeframe?
b) Can you identify any difficulties that the applicants are likely to encounter in meeting their milestones?
c) Have the major ethical, scientific, technical and organisational challenges, as well as any issues around intellectual property rights, been identified and will they be addressed adequately?
d) Are the necessary clinical, academic or organisational links needed to support the research, or help translate it into practice, in place?
e) Is long-term, large scale programme grant funding appropriate; is there added value over and above the dividends from the individual elements?

Management arrangements and timescale are clear and achievable. I have raised a number of points above that will have implications for the successful delivery of the work e.g. ethics

concerning recruitment at the point of crisis; operationalization of key concepts such as ‘crisis’ and ‘successful implementation’; paucity of information about the qualitative work in particular.

One risk identified by the team is of unforeseen national initiatives impacting on the delivery of the intervention. This is indeed a very major risk. It is hard to know how best to address this in a research proposal, but to mitigate by 'raising with NIHR' is probably inadequate!

---

3. STRENGTH of the research team

a) Are the applicants familiar with the methodologies outlined in their application and are they well qualified to undertake the proposed work on the basis of track record in relevant areas (as judged by publication output and previous research funding)?
b) Does the research team have the necessary breadth and depth of expertise to deliver the planned work?
c) Are the roles of the team members clearly described, and is the overall team well coordinated?
d) If the lead applicant is inexperienced, does he/she have appropriate support (e.g. from their host institution and more senior colleagues) to deliver the work plan?

This is very experienced research team and the composition of the team covers the necessary breadth of expertise. It is a very large team, most applicants having a very minimal time contribution to the project, so will need very strong management arrangements. However, I am sure this will be achieved by the team. The team does include a lay member as a co-applicant.

---

4. IMPACT of the proposed work

a) Have the applicants outlined an appropriate and adequate approach to disseminating the result of their research (including engaging with healthcare planners and/or policy makers)? Could this be developed further?
b) Considering the plans for dissemination, what is the likelihood of a significant benefit to the NHS and patients within 3-5 years of the end of the funding period?
c) Have the applicants identified any new intellectual property that will be produced during the course of this research and are there plans to protect and exploit it?

These plans are satisfactory.

The protocol may have implications for intellectual property but this has been considered by the team and initial discussions held with relevant groups.

---

5. VALUE for money (justification for proposed costs)

a) Are the resources required for this research, including staffing, clearly justified? Are they essential for the work proposed?
b) Where the application offers arguments about financial benefits, please assess how realistic these might be.
c) Taking into account the expected benefits of the work proposed and the level of resources requested, does this application represent good value for money?
d) If required, are funds requested for Support and Treatment Costs appropriate?

The actual costing of the bid are satisfactory. I am unsure that it represents good value for money given that there has already been a lot of (funded) development work on the intervention and that there are some weaknesses (as above) in the design. That said, such intervention teams promise significant economic (and psycho-social health) gains for those in distress and at high risk of admission to hospital.

---

6. INVOLVEMENT of patients and the public

Where applicable (N.B. if there is no patient and public involvement, please address question d below)
a) What is your assessment of the patient and public involvement (if any) in the development of the application including involvement in: identifying the research topic; prioritising the research questions; preparing the application (e.g. contributing to the research design)?
b) What is your assessment of any proposed plans for patient and public involvement throughout

the duration of the research? Can you identify particular strengths, weaknesses and/or areas for improvement?
c) Are the resources set aside for patient and public involvement appropriate - including plans for a training and support budget?
d) If there is no patient and public involvement in the application, what is your assessment of the reasons given for this?

PPI appears to be strong. There has been significant involvement already in scoping the content of the proposed intervention (the Home Treatment Protocol). Other ways in which there is PPI are: an ex-carer is a co-applicant; 6 monthly PPI steering group; working alongside DenDRoN, Dementia UK, Age Concern and Alzheimer's Society.

---

7. Additional comments

Do you have any other comments or suggestions for how the proposed research might be improved? If so, please indicate whether you see these as critical factors.

I have raised several areas above that would benefit the overall study if they were adddressed. I do think this is achievable.

PGfAR Competition 13 Stage 2

Peer Review Form

**Application Reference**	RP-PG-0612-20004
**Application Title**	Achieving Quality and Effectiveness in Dementia Using Crisis Teams (AQUEDUCT)
**Applicant**	Professor Martin Orrell
**Applicant Organisation**	University College London
**Total Amount Requested**	£1,978,647.00
**Reviewer Number**	9

**Reviewer Expertise**	Researcher in the same/a very similar field<br>Stroke care and rehabilitation; Dementia care; Palliative care

1. RELEVANCE of the proposed research

a) How relevant and important is the proposed research to the priorities and needs of the NHS, and does it offer a health/healthcare solution with demonstrable benefit to patients?
b) Does the application demonstrate an awareness and understanding of previous relevant research or developments in this area?
c) To what extent does the proposed research add distinct value or provide an advance to what is already known from other work currently in progress or already completed in this area?

There is little doubt that this proposal addresses an important clinical problem. There is also little doubt that the experiences of people with dementia and their carers may well experience 'muddled' services in times of crisis. I do, however, feel that there remains a number of questions about the nature of the intervention proposed and the environments into which this will be delivered and evaluated.

2. QUALITY of the proposed work

2a. Research design

a) Is the proposed research of high quality and does it address the stated objectives?
b) How convincing and coherent is the proposed rationale and approach?
c) Is the proposed design and methodology for all elements of the research well defined, appropriate, valid, robust and feasible within the timeframe and resources requested?
d) What are the strengths and weaknesses of the research design as proposed?

The team has clearly considered very carefully the feedback delivered at an earlier stage. I do, however, feel that there remain concerns about the nature of the intervention itself. It is not clear how existing knowledge and practice will contribute to the nature of the RK. The HTP continues to provide the only major component of the intervention so far developed and I would be concerned that the team may struggle to identify additional components to form a substantial and novel RK.
I understand that there are teams within NHS Trusts charged with crisis work of this nature, but there is little evidence here that the team understand the range and diversity of provision of this

nature. Hence, there is little insight into the type, structure and culture of environments into which the intervention would be expected to work.
I have been asked to review qualitative aspects of the study in particular and I feel that plans are poorly defined. early phases of the work suggests and ethnographic element of the work, but there is limited description about how data will be collected and analysed. The term 'ethnographic analysis' is used but I am not sure what is meant by this term. The use of framework analysis is also used, but little rationale for its use here. In addition there is little consideration of the challenges for the involvement of people with dementia in this type of data collection activity.

---

2. QUALITY of the proposed work

2b. Work plan and proposed management arrangements

a) Do the work plan and project management arrangements give confidence that proposed milestones will be met within the specified timeframe?
b) Can you identify any difficulties that the applicants are likely to encounter in meeting their milestones?
c) Have the major ethical, scientific, technical and organisational challenges, as well as any issues around intellectual property rights, been identified and will they be addressed adequately?
d) Are the necessary clinical, academic or organisational links needed to support the research, or help translate it into practice, in place?
e) Is long-term, large scale programme grant funding appropriate; is there added value over and above the dividends from the individual elements?

I think that the main problem here is that we do not have sufficient knowledge of the type of teams the intervention would be expected to reside. I think that systematic and comprehensive understanding about the nature of such teams would need to be conducted prior to a consideration of this type of intervention. There appear good organisational links between academic and clinical networks and the plans for management of the project appear sound.

---

3. STRENGTH of the research team

a) Are the applicants familiar with the methodologies outlined in their application and are they well qualified to undertake the proposed work on the basis of track record in relevant areas (as judged by publication output and previous research funding)?
b) Does the research team have the necessary breadth and depth of expertise to deliver the planned work?
c) Are the roles of the team members clearly described, and is the overall team well coordinated?
d) If the lead applicant is inexperienced, does he/she have appropriate support (e.g. from their host institution and more senior colleagues) to deliver the work plan?

The team has demonstrated excellence in this field and I would have every confidence that the project would be delivered. I do, however, feel that the team lacks a senior lead for the qualitative aspects of the study and recommend that the team address this. Above and beyond this problem there appears a range of skills and expertise.

---

4. IMPACT of the proposed work

a) Have the applicants outlined an appropriate and adequate approach to disseminating the result of their research (including engaging with healthcare planners and/or policy makers)? Could this be developed further?
b) Considering the plans for dissemination, what is the likelihood of a significant benefit to the NHS and patients within 3-5 years of the end of the funding period?
c) Have the applicants identified any new intellectual property that will be produced during the course of this research and are there plans to protect and exploit it?

I think given the concerns expressed above I would find it difficult to assess the impact of the project favourably. It is clear that I consider this proposal to be at a very early stage in terms of the development of an intervention, above and beyond the HTP.

---

5. VALUE for money (justification for proposed costs)

a) Are the resources required for this research, including staffing, clearly justified? Are they

essential for the work proposed?
b) Where the application offers arguments about financial benefits, please assess how realistic these might be.
c) Taking into account the expected benefits of the work proposed and the level of resources requested, does this application represent good value for money?
d) If required, are funds requested for Support and Treatment Costs appropriate?

The plans appear sound and all costs are appropriately justified.

---

6. INVOLVEMENT of patients and the public

Where applicable (N.B. if there is no patient and public involvement, please address question d below)
a) What is your assessment of the patient and public involvement (if any) in the development of the application including involvement in: identifying the research topic; prioritising the research questions; preparing the application (e.g. contributing to the research design)?
b) What is your assessment of any proposed plans for patient and public involvement throughout the duration of the research? Can you identify particular strengths, weaknesses and/or areas for improvement?
c) Are the resources set aside for patient and public involvement appropriate - including plans for a training and support budget?
d) If there is no patient and public involvement in the application, what is your assessment of the reasons given for this?

There is some evidence that the team has consulted with people with dementia and their carers and it appears that the team also has sufficient plans in involving people with dementia and their carers in further aspects of the study. This is challenging work and despite prior experience, not additional strategies aimed at maximising the involvement of people with dementia are outlined.

---

7. Additional comments

Do you have any other comments or suggestions for how the proposed research might be improved? If so, please indicate whether you see these as critical factors.
